# Dynamic motor practice improves movement accuracy, force control and leads to increased corticospinal excitability compared to isometric motor practice

**DOI:** 10.3389/fnhum.2022.1019729

**Published:** 2023-01-05

**Authors:** Malene Norup, Jonas Rud Bjørndal, August Lomholt Nielsen, Patrick Wiegel, Jesper Lundbye-Jensen

**Affiliations:** ^1^Department of Nutrition, Exercise & Sports, University of Copenhagen, Copenhagen, Denmark; ^2^Department of Midwifery, Physiotherapy, Occupational Therapy and Psychomotor Therapy, Faculty of Health, University College Copenhagen, Copenhagen, Denmark

**Keywords:** motor learning, position control, force control, corticospinal excitability, visual feedback

## Abstract

The central nervous system has a remarkable ability to plan motor actions, to predict and monitor the sensory consequences during and following motor actions and integrate these into future actions. Numerous studies investigating human motor learning have employed tasks involving either force control during isometric contractions or position control during dynamic tasks. To our knowledge, it remains to be elucidated how motor practice with an emphasis on position control influences force control and vice versa. Furthermore, it remains unexplored whether these distinct types of motor practice are accompanied by differential effects on corticospinal excitability. In this study, we tested motor accuracy and effects of motor practice in a force or position control task allowing wrist flexions of the non-dominant hand in the absence of online visual feedback. For each trial, motor performance was quantified as errors (pixels) between the displayed target and the movement endpoint. In the main experiment, 46 young adults were randomized into three groups: position control motor practice (PC), force control motor practice (FC), and a resting control group (CON). Following assessment of baseline motor performance in the position and force control tasks, intervention groups performed motor practice with, augmented visual feedback on performance. Motor performance in both tasks was assessed following motor practice. In a supplementary experiment, measures of corticospinal excitability were obtained in twenty additional participants by application of transcranial magnetic stimulation to the primary motor cortex hot spot of the flexor carpi radialis muscle before and following either position or force control motor practice. Following motor practice, accuracy in the position task improved significantly more for PC compared to FC and CON. For the force control task, both the PC and FC group improved more compared to CON. The two types of motor practice thus led to distinct effects including positive between-task transfer accompanying dynamic motor practice The results of the supplementary study demonstrated an increase in corticospinal excitability following dynamic motor practice compared to isometric motor practice. In conclusion, dynamic motor practice improves movement accuracy, and force control and leads to increased corticospinal excitability compared to isometric motor practice.

## Introduction

Motor performance and learning are essential in our everyday life and we need to optimize motor control, acquire new motor skills and maintain acquired skills throughout our lifespan (Clark, 2007; Beck et al., 2021a,b). When we perform a movement, both proprioceptive, cutaneous, and visual feedback are important for our execution, and humans with intact sensation can weigh the sensory inputs from different sensory modalities to inform our motor control and meet task-specific demands (see e.g., Scott, 2004, 2012). When executing a movement, our force and position control must fit the intended movement and thus the predicted outcome. When this is not the case, the comparison between the motor plan, and the predicted and experienced sensory feedback can be used to adjust our errors and refine our movements across time (Wolpert et al., 2011). Consequently, we learn to graduate the force of our muscle contractions and the position of the limbs, we use for the specific movement, through motor practice.

During recent decades, several studies have investigated principles of motor learning and the involved mechanisms, and several diverse types of motor learning and multiple different processes contribute to our ability to improve our motor abilities with practice (Spampinato and Celnik, 2021). While laboratory tasks to study motor control and motor learning can occasionally seem contrived (Seidler and Carson, 2017), different learning paradigms do have relevance to investigating specific aspects of learning and the involved mechanisms.

The majority of motor learning studies in humans have focused on adaptation and sequence learning and while these paradigms investigate important aspects of learning, they do not necessarily involve skill learning or challenge motor execution (Krakauer et al., 2019). Few studies have focused on ballistic motor learning in which movements cannot already be executed at ceiling levels (see e.g., Muellbacher et al., 2002; Lundbye-Jensen et al., 2011; Roig et al., 2014; Yamaguchi et al., 2020a). Ballistic motor learning studies have e.g., demonstrated the involvement of the primary motor cortex in motor memory consolidation processes, but the behavioral paradigm does not involve aspects of motor acuity. In this manuscript, we focus on motor skill learning and the ability to improve motor acuity with practice.

In recent years, several studies have used a variety of behavioral models with different task characteristics to study human motor skill learning without perturbations. These studies often involve visuomotor target tracking tasks as in the arc pointing task (see e.g., Shmuelof et al., 2012, 2014) and may involve elements of sequence learning as in the sequential visual isometric pinch task, SVIPT (see Reis et al., 2009; Statton et al., 2015). More importantly, the main focus of these tasks is on motor skill learning and acuity development defined as shifts in the speed-accuracy tradeoff function with motor practice (Shmuelof et al., 2012).

Different studies have focused on learning models adapted for finger and pinch tasks, hand and wrist tasks, ankle joint tasks, etc. without perturbations. One interesting observation is, however, that while some studies investigate the effects of dynamic motor practice involving actual movement other skill learning studies use behavioral models involving force control during isometric contractions. This is the case for finger and pinch tasks where, e.g., Reis et al. (2009) and Beck et al. (2020) have investigated the effects of isometric pinch tracking while, e.g., Cirillo et al. (2011), Larsen et al. (2016), and Christiansen et al. (2017, 2020) have investigated effects of the dynamic practice of finger movements. For the wrist, Shmuelof et al. (2012, 2014) investigated the effects of dynamic wrist movements in the arc pointing task. The participants in this study trained wrist movements at different movement times; moderate, fast, and slow compared to a resting control group. The performance measure was the speed-accuracy-function and variability of movement trajectories. Similarly, Wiegel and Leukel (2020) also used the practice of dynamic wrist flexions to investigate effects on corticospinal excitability, while Roig et al. (2012) and Thomas et al., 2016a,b used isometric wrist force tracking to investigate changes in movement accuracy with motor practice and effects of exercise on motor memory. Collectively, both dynamic and isometric motor learning paradigms have been employed in the research literature to investigate overlapping research questions relating to motor learning, consolidation processes, and retention effect in addition to investigating neurophysiological effects accompanying motor control and practice e.g., changes in the primary motor cortex and corticospinal pathway (e.g., Perez et al., 2006; Christiansen et al., 2018, 2020, etc.).

While multiple studies have demonstrated the effects of dynamic motor practice on movement accuracy and precision and the effects of isometric motor practice on force control, very few studies have to the best of our knowledge compared behavioral and neurophysiological effects of dynamic and isometric motor practice. In the present study, we, therefore, aimed to compare the effects of dynamic motor learning and isometric motor learning on both wrist position control and wrist force control. Hence, we also aimed to assess whether dynamic motor practice leads to improvements in force control and conversely whether isometric motor practice leads to improvements in position control in young adults.

We hypothesized that dynamic motor practice without online eye-hand coordination but with augmented feedback leads to improved position control. Similarly, we also expected that isometric motor practice leads to improved force control. Secondarily, we hypothesized that both types of motor practice will have a positive effect on performance in the non-practiced domain, but that position control motor practice has a greater effect on force control than force control motor practice will have on position control since dynamic movements can also involve mechanisms relating to force control. We addressed these questions in an experimental design where participants either practiced a session of position control (PC), Force control (FC), or rested (CON). Before and after this, performance in both tasks was tested in a counterbalanced order.

Furthermore, in a supplementary experiment, we aimed to investigate whether dynamic vs. isometric practice of an otherwise identical task has differential effects on corticospinal excitability. Here we expected motor practice to be accompanied by increased corticospinal excitability and we hypothesized that this effect would be more pronounced following position control motor practice compared to force control motor practice.

## Materials and methods

### Participants

Sixty-eight young adults (20–30 years) (46 participants in the main experiment, and 20 participants in the supplementary experiment) were recruited from the Copenhagen area to participate in the study. All participants were naïve to the motor tasks used in the study. Participants were included based on age, and general health, and a general eligibility questionnaire was used for this inclusion procedure. Participants had no history of neurological or psychiatric diseases, no intake of medication, and had a normal or corrected-to-normal vision. To ensure that the prerequisites according to difficulty in the motor task were as even as possible handedness was assessed by use of the Edinburgh Handedness Inventory (Oldfield, 1971), according to which the participants were right-handed. Additionally, all participants completed questionnaires on sleep the night prior to the experiment and sleepiness during the experiment (Hoddes et al., 1973). For descriptive information on the participant characteristics, see Tables 1, 2. All experimental procedures were approved by the regional ethics committee for the Greater Copenhagen area (H-17019671) and the study was performed in accordance with the declaration of Helsinki. All participants received written and oral information about the experimental procedures and gave their written informed consent prior to participating in the study.

**TABLE 1:** Main experiment: Characteristics of participants.

Intervention group	Position control (PC)	Force control (FC)	Resting control (CON)	*P*-values range
Sex (m/w)	15 (7/8)	16 (6/10)	15 (6/9)	
Age (years)	25.2 ± 2.2	24.7 ± 1.9	24.5 ± 2.7	0.4–0.7
Weight (kg)	74.7 ± 15.9	66.2 ± 17.0	70.1 ± 10.6	0.2–0.6
Height (m)	1.77 ± 0.09	1.69 ± 0.11	1.74 ± 0.08	0.1–0.3
Handedness (LQ)	81.5 ± 27	90.8 ± 10.4	87.3 ± 12.9	0.3–0.6
Sleep (h)	8.0 ± 0.7	7.9 ± 1.3	7.3 ± 1.2	0.1–0.7
Sleepiness (SSS, 1–5)	2.1 ± 0.8	2 ± 0.8	2.7 ± 1	0.1–0.4
MVC (N)	73.9 ± 47.8	74.7 ± 43.9	68.9 ± 37.3	0.7–1.0

Data reported as means ± SD. LQ, laterality quotient; Sleep, hours slept night before the experiment; SSS, Stanford sleepiness scale, assessment obtained during experiment. *T*-tests used to test for between-group differences at baseline.

**TABLE 2:** Supplementary experiment: Characteristics of participants.

Group	Position control (PC)	Force control (FC)	*P*-value
Sex (male/female)	10 (8/2)	10 (8/2)	
Age (years)	24.4 ± 2.1	24.5 ± 2.3	0.9
Handedness (LQ)	86.5 ± 11.8	89.5 ± 12.3	0.6
Sleep (h)	8 ± 1	7.8 ± 1.2	0.8
Sleepiness (SSS, 1–8)	2.4 ± 0.5	2.5 ± 0.5	0.7

Data reported as means ± SD. LQ, laterality quotient; Sleep, hours slept the night before the experiment; Sleepiness, Stanford sleepiness scale, assessment obtained during experiment. *T*-test used to test for between-group differences at baseline.

### Study design

**FIGURE 1:**
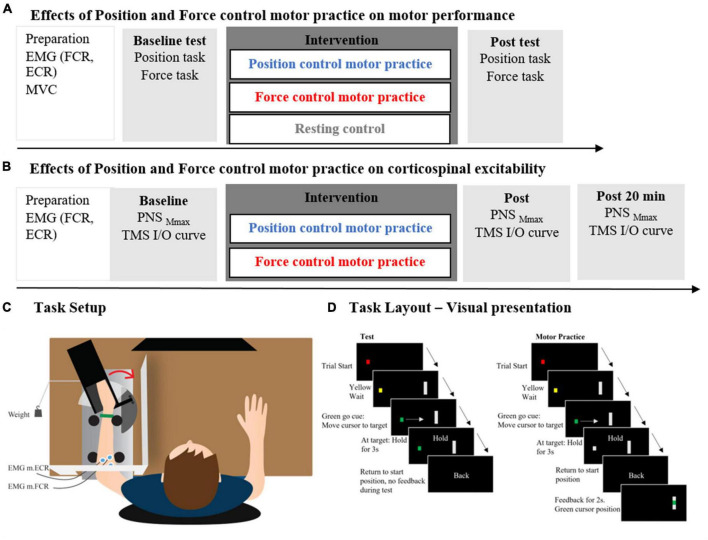
Panel **(A)** depicts the experimental protocol of the main experiments including the two intervention groups: position control practice and force control practice and the control group. In panel **(B)** the experimental protocol of the supplementary experiments is depicted including the two intervention groups: position control practice and force control practice. The experimental setup is shown in panel **(C)** while the computerized task is shown in panel **(D)**.

The main experiment consisted of a between-group design with 46 participants randomized into three groups with different motor practice conditions: (1) Position control practice group (PC), (2) force control motor practice group (FC) and (3) a resting control group (CON) (Figure 1A). Participants were randomized to one of the three groups after balancing for sex (m/f). Assessors were not blinded to the group allocation. Measures of sensorimotor performance were obtained in both a wrist force control task and a wrist position control task at baseline (prior to motor practice) and following motor practice. This experimental design allowed us to investigate the acute effects of sensorimotor training with the emphasis on position and force control, respectively, on accuracy in both a force and position control task, and to assess possible transfer effects between tasks.

In the supplementary experiment, measures of corticospinal excitability were obtained in twenty additional participants by application of transcranial magnetic stimulation (TMS) to the primary motor cortex hot spot of the flexor carpi radialis muscle before either position or force control motor practice. TMS measures were obtained again immediately after motor practice and 20 min later (Figure 1B).

### Experimental setup

During all experiments, participants were seated in a chair in front of a computer monitor (27”, Lenovo Thinkvision, resolution 2,550×1,440 pixels). The left forearm was placed horizontally in a semi-prone position in a custom-molded armrest in front of the participant and the height was adjusted for the shoulder to be in a neutral position. This meant that the shoulder was in a position of slight shoulder abduction and the elbow joint flexed to approximately 100°. The left hand was placed in a custom 3D printed box with fingers passively extended. The box was mounted on a lever, which was able to rotate around the same axis as the movements of the wrist joint. The forearm was fixated with Velcro bands and the entire setup allowed wrist movements specifically. The setup had a built-in strain gauge and goniometer, which allowed measurement of wrist position and—when the handle was fixed—wrist flexion force. To prevent direct visual eye-hand feedback, a custom-built white screen hid the left arm during all motor tasks and motor practices. The right arm was resting on the table in front of the participant (Figure 1C).

### Motor tasks

During the experimental sessions, participants performed wrist flexion movements in two different motor tasks (inspired by Wiegel and Leukel, 2020 and Wiegel et al., 2022). The handle was either (1) fixated to allow force control during isometric wrist flexions or (2) to allow dynamic position control during the position control task. For both the force control task and the position control task, the initial starting position was a 40° extension relative to the neutral position (*in situ*). The computerized position- and force tasks consisted of a black screen showing a gray vertical line (indicating a target) and a “traffic light” indicating preparation (yellow dot) and the start signal of the movement (green dot). A red dot indicated the start and end of a trial (custom-made scripts created for the purpose in MATLAB R2019a, MathWorks Inc.). When the dot turned green the participant had to reach the target within 1 s. Hereafter, “Hold” was displayed on the screen for 3 s informing the participants to hold the current position/force level as accurately as possible. The last message on the screen was “Back,” indicating the end of the trial and return to starting position. This was followed by the red dot on the screen, meaning a new trial is upcoming (Figure 1D). All movements on the screen were in the horizontal plane.

During the position control tasks, the handle was connected to a wire holding an individually adjusted weight (100–400 g) to ensure that the position of the hand returned to the starting position by pulling the wire and thereby returning the hand to the starting position after the hold phase. The position control test consisted of a series of discrete movements to five predefined target positions. Target positions were at 15°, 30°, 45°, 60°, and 75° flexion relative to the starting position at 40° degrees dorsal flexions.

In the force control task, the handle was fixated in the starting position to allow measurements of isometric wrist force. The force control test consisted of a series of 40 isometric contractions in the starting position at different percentages of maximal voluntary contraction (MVC): 5, 10, 15, 20, and 25%.

For both tasks targets, 1–5 were placed with 400 pixels horizontal displacement between targets. This means that target 5 (i.e., 75° flexion movement or 25% MVC) required 2,000 pixels of horizontal cursor displacement. The edge of the monitor (2,550 pixels) corresponded to 90° flexion or 30% MVC.

### Protocol for the main experiment and the supplementary experiment

During the main experiment, participants were introduced to a maximal voluntary contraction test (MVC) for wrist flexion via verbal instructions and a PowerPoint presentation illustrating the test. Participants were instructed to push with the palm and only to palmar flex the wrist to avoid contraction of other arm and shoulder muscles. Participants attempted to reach maximal force within 3 s and received verbal encouragement during the trials. The peak force value obtained in the best of the three trials was identified as MVC and used to calculate target positions in the force task.

After the MVC test, baseline performance in the force and position task was assessed. The order of these tests was counterbalanced between participants in all three groups. Both tests were preceded by an introduction (visual and verbal) and familiarization with the task. Instructions were provided as a visual presentation and preceded each test. Following instructions, the participants were allowed three familiarization trials for each of the first, middle, and last target positions. This was repeated before each test both before and after motor practice. During the familiarization trials, a green cursor was visible during the movements/contractions from the starting position to the endpoint.

Following instruction and familiarization with the position control and force control tasks, participants performed a baseline test in both tasks with no augmented feedback on performance i.e., only the target position was displayed.

During the sensorimotor practice interventions in the main and the supplementary experiments, participants completed four blocks of 40 trials of either the position or force task depending on the intervention group. During the four practice blocks, the end position of the green cursor was visible after each movement to provide augmented feedback on the position or force level, respectively. The endpoint was the average position/force level calculated during the “Hold” period. Additionally, verbal encouragement (“Good job,” “Well done,” “Keep up the good work”) was provided approximately every 10th trial to ensure motivation throughout the practice session. The control group in the main experiment rested for 30 min in a seated position similar to that of the participants performing motor practice.

Following motor practice in the main experiment or rest as in the control condition, performance in the position and force control tasks was assessed again following the same procedure as described for the baseline tests including instruction and familiarization before the actual tests consisting of 40 trials in the force control task and 40 trials in the position control task. To assess the sleepiness of the participants during the experiment, they filled out the Stanford sleepiness scale before the behavioral tests (Hoddes et al., 1973).

For the supplementary experiment, the same procedures were followed for motor practice as in the main experiment i.e., participants practiced either the position or force control task 4 blocks of 40 trials. In the supplementary experiment no behavioral measures were obtained prior to or following motor practice. Instead, eletrophysiological tests involving transcranial magnetic stimulation and peripheral nerve stimulation were performed. These procedures are described in the following paragraphs.

### Recording and stimulation procedures

During the experiment, wrist force and position signals were amplified, low-pass filtered (10 Hz) A/D-converted, and sampled at 1 kHz in Matlab 2019A (Mathworks, US) for the position and force control task. Additionally, all signals were sampled on a computer for offline analysis at 2,048 Hz with Signal Software v7.5 (Cambridge Electronics Design, UK). During the main experiment, electromyography (EMG) was recorded from the FCR and *extensor carpi radialis* (ECR) muscles of participants’ left arm through surface electrodes (Biosemi, NL) applied on the skin after preparation with medical sandpaper. The electrodes were placed in a bipolar muscle-belly—muscle-belly montage with a 2 cm interelectrode distance. During the main experiment, participants were also equipped with an EEG cap with 64 active channels placed according to the international 10–20 system. Both EMG and EEG were sampled as raw signals using the ActiView software (v 7.07) with a sampling rate of 2,048 Hz. The EMG and EEG measures obtained during the main experiment are not analyzed for this manuscript.

In the supplementary experiment, the effects of PC and FC on corticospinal excitability were measured using transcranial magnetic stimulation (TMS). Corticospinal excitability was assessed through recruitment curves obtained with single-pulse TMS (see Christiansen et al., 2018 for corresponding procedure). TMS was applied to the contralateral (right) hemisphere primary motor cortex (M1) by a Magstim Rapid^2^ stimulator (Magstim Company Ltd., Whitland, UK) via a custom made 90 mm Figure-of-eight coil (batwing design, Magstim Company Ltd., Whitland, UK) with the capability to deliver a magnetic field of 2 T. During these experiments, EMG was obtained from the FCR muscle. The EMG was Band-pass filtered (5 Hz–1 kHz) and sampled at 2,048 Hz in Signal Software v7.5 (Cambridge Electronics Design, UK). The optimal coil position (hotspot) for eliciting motor evoked potentials (MEPs) in the FCR muscle was established through a standardized stimulation procedure with high spatial resolution covering the primary motor cortex (M1), i.e., a mapping procedure, at each test (see also Christiansen et al., 2018 for procedure). During the assessment of the resting motor threshold (rMT) and generation of the recruitment curves, the coil was placed with the center oriented parallel to the scalp over the hot spot of the FCR representation with the handle of the coil pointing backward at an angle of 45° to the sagittal and horizontal axis. TMS recruitment curves were obtained by delivering 80 single pulse stimuli in a random sequence with an inter-stimulus interval of 4 s and stimulus intensities ranging from 80 to 170% rMT (Devanne et al., 1997). The rMT was defined as the minimum intensity required to elicit a peak-to-peak MEP amplitude larger than 50 μV in three out of five trials (always below). All TMS measurements were obtained while the participant was at rest. Trials in which any background activity larger than 2 × standard deviations was observed were discarded. A maximum of four trials were discarded from each recruitment curve. During all experiments involving TMS, frameless stereotaxy (Brainsight 2, Rogue Research, Montreal, Canada) was used to identify the coordinates of the M1 hotspot and to monitor the position of the coil relative to the participants’ heads throughout the experiment.

Before the generation of recruitment curves at each test, maximal compound muscle action potentials of the FCR muscle (maximal M-waves, M_max_) were elicited by 1 ms bipolar electrical stimulation of the median nerve above the elbow joint (DS7A constant current stimulator, Digitimer Ltd., UK). Stimulations were applied at 4 s intervals, and the intensity of the stimulation was increased from a subliminal level until there was no further increase in the peak-to-peak amplitude of the M-wave with increasing intensity.

### Data processing and statistical analysis

For the descriptive characteristics of the participants, potential between-group differences were assessed by the use of *t*-tests. Data from the sensorimotor wrist task obtained during baseline and posttests as well as during motor practice was binned in trials of five and included one trial for each target in each data point. Data were transformed to z-scores to identify potential outlier data points based on ± 3 SD (0.5% of Position data removed, and 0.6% of Force data removed).

For the electrophysiological measurements obtained in the supplementary experiment, all motor evoked potentials were quantified as peak-to-peak amplitudes in the raw EMG. This procedure was also performed for the analysis of M-waves obtained in response to peripheral nerve stimulation to identify M_max_. For TMS data, the mean amplitude obtained at each of the stimulation intensities (80–170%) was calculated for each participant at each time point (baseline, post, and 20 min post motor practice). TMS recruitment curves were constructed by plotting the relationship between stimulus intensity and mean MEP peak-to-peak magnitude. Finally, the sum of evoked MEP magnitudes across the recruitment curve was calculated. For further procedures on modeling TMS recruitment curves (see Devanne et al., 1997; Carson et al., 2013; Christiansen et al., 2017, 2020).

All statistical analyses were performed using R (R Core Team, 2022), with the R-package (Bates et al., 2015) used to fit linear mixed effects models to the averaged data points. For both the force and position task, we modeled dependent variables [performance scores: error (pixels), standard deviation, and coefficient of variance (%)] with INTERVENTION (PC, FC, CON) and the repeated measure BLOCK (Baseline, Post practice) as the independent fixed variables, we also included the interaction term (INTERVENTION x BLOCK). To account for inter-individual differences in performance we added intercepts for each participant as a random effect, this was also done for trials within each block. Similar models were used to test for performance gains during practice with the BLOCK condition (practice blocks 1–4). Assumptions of normality and homogeneity of variance of residuals were checked with QQ-plots and residual plots. The *multcomp* R-package (Hothorn et al., 2008) was used for pairwise comparisons based on our hypotheses. The Holm-Sidak method was used to adjust for multiple statistical comparisons. For all statistical analyses, the significance level was set at a *p* < 0.05. Model estimates are presented with standard error (SE) and confidence intervals (CI) when appropriate. Otherwise, tables and results are presented as means and standard deviations for all intervention groups. To explore whether changes in motor performance (error) during motor practice were related to changes in CSE in the supplementary experiment, the error during the first twenty trials of training and the last twenty trials of training was quantified. Correspondingly, change in CSE from pre to post motor practice was also quantified for each participant. Potential correlations were tested with a Pearson correlation for position control motor practice and force control motor practice, respectively.

## Results

Descriptive characteristics of the participants in the main experiment are presented in Table 1, and from the supplementary experiment in Table 2. Forty-eight participants were recruited for the main experiment, of which two were excluded due to technical difficulties during experiments. No differences were observed between groups in descriptive characteristics (Table 1).

### Motor performance in the position task (error)

**FIGURE 2:**
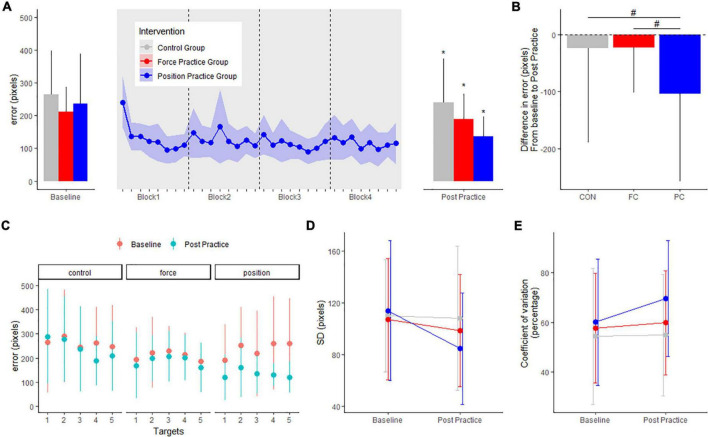
Mean errors (pixels) at baseline and post practice conditions from in the position control task in the three groups in the main experiment are depicted in panel **(A)**: the position control practice group (blue), force control practice group (red) and control group (gray). Learning curves from the four blocks of position control practice is shown in panel **(A)**. The delta changes (error) in the position control task are shown in panel **(B)**. Target specific mean errors (pixels) in the position control task at baseline and post practice in the three groups are shown in panel **(C)**. Standard deviations from mean error in the three groups are shown in panel **(D)** while coefficient of variation in percentage are shown in panel **(E)**. *Denotes a significant difference within group and # denotes significant difference between groups.

For the position task, motor performance is displayed in Figure 2A. There were no differences in baseline error (in pixels) or SD (pixels) between any of the intervention groups PC: 236.5 ± 155.3, FC: 208.7 ± 79.3, CON: 263.1 ± 135.1 pixels (range of *p*-values: 0.1–1). All intervention groups decreased their error from baseline to the post measurement. The PC group, practicing the position task, improved significantly more in the position task compared to both the FC group and CON group, evident from significant between-group differences in the change in error from pre to post (*p* < 0.001) (see Figure 2B). The PC group demonstrated a significant decrease in error (−102.6 ± 7.9 pixels, CI: [−118.1; −87.1], *p* < 0.001), and the FC and CON group demonstrated smaller but also significant decrease in error (FC: −21.8 ± 7.6, CI: [−36.7; −6.8], *p* = 0.004; CON: −22.6 ± 7.9, CI: [−38.1; −7.1], *p* = 0.004).

When investigating the effects of practice on performance for individual targets in the position task, the mean error decreased significantly for all five targets in the PC group (all *p*-values < 0.001). Following FC practice, no significant changes were found for any specific targets from baseline to post (*P*-value > 0.578). The CON group improved significantly only for target 4 (Target 1: *P* = 0.5536. Target 2 *P* = 0.9, Target 3: *P* = 0.9, Target 4: *P* < 0.001. Target 5: *P* = 0.075) (Figure 2C).

We also quantified the standard deviation of the error for each individual at baseline and the post test. In the PC group, the standard deviation between trials decreased significantly more compared to the CON group evident from significant between-group differences in the change in SD from pre to post, but not compared to FC, and no difference between CON and FC was observed (PC vs. FC: −20.7 ± 9.5, *p* = 0.06; PC vs. CON: −27.6 ± 9.7, *p* = 0.01, FC vs. CON: −6.8 ± 9.5, *p* = 0.06) (see Figure 2D). To further assess performance variability, we calculated individual coefficient of variation for each target, before being grouped across targets for each intervention group. Coefficient of variation was significantly different from baseline to post-practice in the PC group, but not in either the FC group or the CON group (PC: 9.6 ± 3.5, CI: [2.6; 16.6], *p* = 0.007; FC: 2.1 ± 3.4, CI: [−4.6; 9.8], *p* = 0.53; CON: 0.4 ± 3.5, CI: [−6.5; 7.4], *p* = 0.89). We observed no significant between-group difference in change from baseline to post-practice (range of *p*-values: 0.21–0.74) (Figure 2E).

### Motor performance in the force task (error)

**FIGURE 3:**
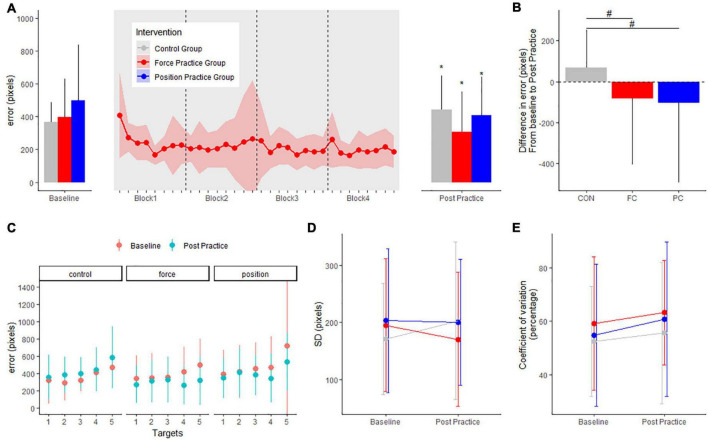
Mean errors (pixels) at baseline and post practice conditions from force control task in the three groups in the main experiment are depicted in panel **(A)**: the position control practice group (blue), force control practice group (red) and control group (gray). Learning curves from the four blocks of force control practice is shown in panel **(A)**. The delta changes (error) in the force control task are shown in panel **(B)**. Target specific mean errors (pixels) in the force control task at baseline and post practice in the three groups are shown in panel **(C)**. Standard deviations from mean error in the three groups are shown in panel **(D)** while coefficient of variation in percentage are shown in panel **(E)**. *Denotes a significant difference within group and # denotes significant difference between groups.

For the force task, motor performance is displayed in Figure 3A. There were no differences in baseline error (in pixels) or SD (pixels) between intervention groups; FC: 392.5 ± 234.3, PC: 494.8 ± 341.7, CON: 365.6 ± 119.1 (range of *p*-values: 0.1–0.9). For both intervention groups error decreased from baseline to post practice while error increased in the control group (FC group: −86.7 ± 15.6, CI: [−117.2; −56.1], *p* < 0.001; PC group: −101.2 ± 16.3, CI: [−133.2; −69.3], *p* < 0.001); CON group: + 68.9 ± 16.3, CI: [36.9; 100.9], *p* < 0.001). Both the PC and FC group improved more in the force task compared to the CON group (*p*-values < 0.001) (Figure 3B).

When investigating the effects of motor practice on individual targets in the force task, the FC group improved significantly for target 4 and target 5 from baseline to post-practice (all *p*-values < 0.001). The PC group improved significantly for targets 3, 4, and 5 from baseline to post-practice (range of *p*-values: 0.001–0.04). The CON group displayed significantly increased errors for targets 2 and 5 from baseline to the post-test (range of *p*-values: 0.001–0.65) (Figure 3C).

Standard deviation between trials did not change from baseline to post practice in any of the intervention groups (PC: −2.5 ± 16.6, CI: [−35.1; 30.1], *p* = 0.88; FC: −22.4 ± 16.1, CI: [−54.0; 9.1], *p* = 0.16; CON: 31.6 ± 16.7, CI: [−1.2; 64.5], *p* = 0.059) (Figure 3D). No significant difference was observed in coefficient of variation from baseline to post practice in any of the groups (PC: 6.0 ± 3.8, CI: [−1.5; 13.6], *p* = 0.11; FC: 3.9 ± 3.7, CI: [−3.4; 11.3], *p* = 0.29; CON: 3.2 ± 3.9, CI: [−4.5; 10.8], *p* = 0.41) (Figure 3E).

### Performance changes between motor practice and test sessions

Endpoint feedback on performance was provided during the practice blocks but not during tests at baseline and post practice. In the PC group, we compared performance levels in the position task from the baseline test to the first block of motor practice. A significant decrease in error was observed from baseline to block 1 (PC: −110.9 ± 7.9, CI: [−110.9; −95.4], *p* < 0.001), this was also seen in the FC group for the force task (FC: −151.0 ± 15.6, CI: [−182.6; −120.5], *p* < 0.001).

Similarly, we compared the performance levels between the last practice block and during the post test. A significant increase in error was observed in the PC group, going from motor practice to the post test (PC: 19.2 ± 7.9, CI: [3.7; 34.7], *p* = 0.015), this was also evident for the FC group in the force task (FC: 110.5 ± 15.3, CI: [80.5; 140.5], *p* < 0.001).

### Effects of test order and feedback

Test order was counterbalanced within all three groups. For the position task planned comparisons showed an effect of the baseline test order. Participants who performed the position task after the force task performed better than those who started with the position task (Position task before Force task vs. Force task before Position task: −118.5 ± 10.6, CI: [−139.7; −98.0], *p* < 0.001). In the force task planned comparisons showed no order effect at baseline (Force task before position task vs. position task before force task: −36.0 ± 21.5, CI: [−36.9; −78.1], *p* = 0.09).

At post practice, none of the groups demonstrated order effects in the position task (range of *p*-values: 0.1–0.9) nor in the force task (range of *p*-values: 0.7–1.0).

Concerning the previously mentioned effect of visual endpoint feedback, we tested for an order effect from the end of motor practice to the order of posttests. In the PC group, error increased as mentioned above from the end of practice to post practice. In detail, error increased significantly when the position task was the first post-test (27.7 ± 8.9, CI: [7.8; 47.7], *p* = 0.003), whereas similar performance levels were seen between the last practice block and the position posttest when the force posttest had come first, evident from non-significant change (1.79 ± 11.7, CI: [−24.3; 27.9], *p* = 0.87). In the FC group, error increased from the end of practice to post practice (110.5 ± 15.3, CI: [80.5; 140.5], *p* < 0.001). Similar increases in error were observed when comparing the last practice block to the first force posttest (119.7 ± 17.3, CI: [81.09; 158.3], *p* < 0.001), or the second force posttest (90.2 ± 23.5, CI: [37.6; 142.7], *p* < 0.001).

### Effects of position and force practice on corticospinal excitability

The supplementary experiment investigated the effects of position and force control motor practice on corticospinal excitability. No differences were observed between the groups in descriptive characteristics (Table 2).

No significant changes were observed in M_max_ amplitudes from baseline to post motor practice. MEP amplitudes were not normalized to M_max_ but reported as raw amplitudes.

**FIGURE 4:**
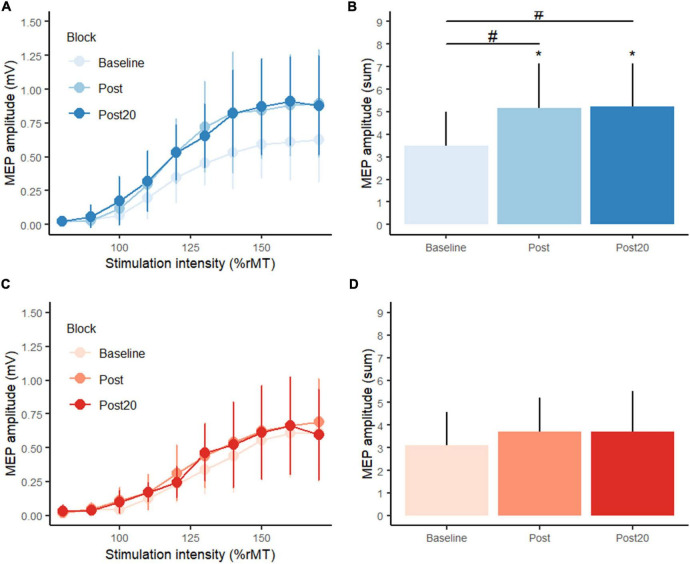
Transcranial magnetic stimulation (TMS) stimulus-response curves from **(A)** the position control practice group (blue colors), **(C)** the force control practice group (red colors) at baseline, post practice and 20 min post practice from the supplementary experiment. Panels **(B,D)** display summed motor evoked potential amplitudes (MEP) at baseline, post practice and 20 min post practice. *Denotes a significant difference within group and # denotes significant difference between groups.

Considering corticospinal excitability, planned comparisons showed no difference between-group at baseline in the sum of MEP amplitudes (*p* = 0.609). See Figure 4. Following position practice, MEP amplitudes increased significantly (*p* = 0.0014), and this was also the case 20 min after position practice compared to baseline (*p* = 0.0014). Following force control practice, while mean MEP values seemingly increased, there was not a significant change (*p* = 0.482) and neither 20 min later (*p* = 0.482). At post practice, the PC group showed significantly higher MEP amplitudes compared to the FC group (*p* = 0.0426) and this difference in corticospinal excitability was still observed 20 min after motor practice (*p* = 0.0351). There were no significant correlations between change in motor performance (error) and changes in CSE (sum of MEP amplitudes) for either type of motor practice.

## Discussion

The present study aimed to investigate the effects of discrete motor task practice with an emphasis on either position control or force control on movement accuracy and force control and to investigate whether the motor practice in the two motor paradigms is accompanied by differential changes in corticospinal excitability in young adult participants.

The results demonstrated that both types of motor practice induced changes in motor performance. These effects were however distinct depending on the type of motor practice. In the following paragraphs, we will discuss the results and their implications.

### Dynamic motor practice with an emphasis on position control leads to improved movement accuracy

The results from the group practicing the reinforcement motor learning task with an emphasis on position control demonstrated that performance in the position task without augmented feedback improved from baseline to post measures with a decrease in the summed error (in pixels) across all targets. The improvement was bigger compared to both the force practice group and the control group, and the result is in line with our hypothesis that the type of motor task practiced will lead to improved performance in the same conceptual task from baseline to post measures even in the absence of augmented feedback. Looking deeper into the performance (error) for the specific targets (i.e., 15°, 30°, 45°, 50°, and 75° wrist flexion), the results demonstrated that dynamic motor practice led to improved position control i.e., reduced error for all target positions.

These findings are in line with previous studies demonstrating behavioral improvements accompanying dynamic motor practice. The task utilized in the present study is similar to the task employed by Wiegel and Leukel (2020). When used dynamically, the task is a discrete spatiotemporal wrist flexion accuracy task. In this previous study, the dynamic motor practice also led to improved motor performance (smaller movement errors) and reduced kinematic variability of the discrete wrist movements (Wiegel and Leukel, 2020). This corresponds to the findings in other studies demonstrating improved movement accuracy following short-term dynamic motor practice (e.g., Larsen et al., 2016; Christiansen et al., 2020, etc.).

Shmuelof et al. (2012) also used dynamic wrist movements to assess the effects of motor skill practice in young adults. The participants in this study trained wrist movements at different movement times; moderate, fast, and slow. The performance measure was the speed-accuracy function (SAF) and variability of movement trajectories. The results showed that training of dynamic wrist movements at moderate movement time (520–780 ms) led to significant improvements in accuracy and a significant decrease in movement time. They also found that, after wrist movement training sessions on 3 consecutive days, the variability of the movement trajectory decreased at all speeds (Shmuelof et al., 2012). This was also found by Shmuelof et al. (2014) where participants also practiced a reinforcement motor task involving movements with a single constraint movement time, but tests were performed at different movement times. The study showed that motor practice in restricted speed ranges led to a global shift of the SAF. Although there were small changes in mean trajectory, improved performance largely consisted of a reduction in trial-to-trial variability and an increase in movement smoothness. When comparing the results of the present study, it seems that dynamic motor practice leads to consistent improvements in movement accuracy. In the present experiments, we did not assess SAF, but the task had a fixed movement timing directed by instructions and visual cues and 1 s to perform the movement before the *hold* period started. Improved accuracy in the dynamic motor task thus did not result from changes in movement speeds.

### Isometric motor practice improves force control

The results from the group of participants practicing force control with augmented feedback demonstrated a positive effect on motor performance in the force task with decreased error across all targets. This improvement was bigger compared to the control group, and the finding supports our hypothesis that isometric motor practice would lead to task or domain-specific improvements in force control. We further investigated behavioral effects for each of the five different targets in the force task following motor practice. We found that force control practice led to improvements i.e., lower error for targets 4 and 5 (20 and 25% MVC) but not for targets 1–3 (i.e., 5, 10, and 15% of MVC). In line with this, Kumar and colleagues previously found that accuracy improved less at smaller force levels (targets) during the initial stages of force task practice depending on which task was performed (Kumar et al., 2017).

Previous studies investigating the effects of isometric motor practice have also found improvements in motor performance during and following training (Yamaguchi et al., 2020b). These studies also involve isometric wrist tasks (Roig et al., 2012, 2014; Thomas et al., 2016a) or isometric pinch tasks involving accuracy tracking (Christiansen et al., 2018; Beck et al., 2020, 2021a,2021b) or the sequential visual isometric pinch (SVIPT) task as employed by e.g., Reis et al. (2009), Statton et al. (2015), and others. It should, however, be noted that while these studies involve motor practice of force control, the tasks all involve online visual tracking of the exerted force. The tasks are in other words all visuomotor force tracking tasks whereas, in the present study, the force task is a discrete task without online feedback. During practice, augmented feedback on force performance was presented. In the majority of previous studies, force control was not assessed without online or augmented feedback following motor practice. The setup in the present study does, however, demonstrate that in the absence of online and augmented feedback, force control is improved for larger force levels following force control motor practice.

### Dynamic motor practice improves force control but not vice versa

Performance in force vs. position control task has been studied previously for different populations (see e.g., Baudry et al., 2010) and Lauber et al. (2012, 2013a) have demonstrated that providing augmented feedback based on joint position vs. force changes motor performance and cortical processing. To our knowledge, it has however, not previously been tested if or to which extent dynamic motor practice with an emphasis on position control leads to changes in (isometric) force control with the same body part or vice versa.

In addition to improvements in position control, the dynamic motor practice also led to improvements in the force control task with a decrease in error (in pixels) from baseline to post-intervention. Looking deeper into the performance for the specific targets, the position control motor practice led to improved performance for targets 3, 4, and 5 (i.e., 15, 20, and 25% MVC) in the force control task. We did not find that the position control practice improved performance at the lowest levels of force (5 and 10% of MVC). In conclusion, dynamic motor practice with an emphasis on position control led to similar changes in force control as those accompanying force control motor practice. Thus, both types of training were accompanied by positive effects on force control compared to the control group with no difference between training groups—although dynamic practice led to improved performance for three out of five force targets and isometric practice led to improved performance for two out of five force targets.

While dynamic motor practice with an emphasis on position control leads to improvements in a force control task, isometric motor practice with an emphasis on force control did not lead to improvements in position control. While the force group improved performance from pre to post across all targets, the improvement was not different from the control group which did not perform motor practice and there were no significant changes in performance for either of the five position targets. Thus, motor practice with an emphasis on force control did not lead to improvements in position control.

Collectively, the results demonstrate that motor practice with an emphasis on position control has positive transfer effects to a force control task, whereas the opposite transfer was not observed.

Both proprioceptive, cutaneous, and visual feedback are important for movement execution and healthy humans with intact sensation are able to weigh the sensory inputs from different sensory modalities in order to inform e.g., position or force control during movements and meet task-specific demands. Additionally, correction of movement errors and successful motor learning often rely on the integration of different types of sensory feedback. Before the experiment, we speculated that since dynamic motor practice involves active movement including proprioceptive feedback, cutaneous inputs from the hand, and during motor practice also visual feedback, performance in the task can involve elements of both position and force control. Since the applied force would expectedly lead to changes in position in the dynamic task it would be meaningful to utilize force control in addition to position control and this may have led to the observed transfer effect. There was no positive effect of isometric motor practice on position control. Previous studies have demonstrated that force control—like position control—relies on monitoring of motor output and integration of sensory feedback including cutaneous feedback (Choi et al., 2013). Whereas, dynamic motor practice may involve aspects of force control, it is however, less likely that proprioceptive feedback can inform position control during an isometric task. Based on this, it could be expected that isometric motor practice has little transfer to a position control task.

We chose not to provide augmented feedback on task performance in the baseline and posttests to minimize learning effects from tests and to allow assessment of behavioral effects post-practice without the influence of augmented feedback including feedback-dependency. This means, that the posttests also act as transfer tests assessing both retention and flexibility of the resultant motor learning (Kantak and Winstein, 2012). We did not have delayed retention tests as part of the design meaning that this does not allow assessment of long-term retention and memory.

Participants did receive augmented feedback (knowledge of performance) for each trial during motor practice and while it is possible that more pronounced behavioral effects could have been observed if successful trials had been followed by augmented feedback as knowledge of result or rewarded e.g., in the way similar to Shmuelof et al. (2014) we nevertheless observed significant effects of practice on performance during learning.

Both at baseline and posttests, the test order was counterbalanced in all three groups meaning that following familiarization, half of the participants started with the position test and the other half started with the force task. At baseline, we found that that participant who performed the force task prior the position task performed better in the position task compared to the opposite order. It is likely that this effect relates to early learning and transfer from the baseline force task test. We did, however, not observe a baseline order effect for performance in the force task meaning that familiarization to the position test did not benefit performance in the force task. This demonstrates that transfer effects are not symmetrical between these two tasks. Following motor practice, we did not observe any test order effects. Other studies have suggested that participants who perform different motor tests following practice may display different degrees of transfer, flexibility or conversely interference effects, and these effect may be influenced by test order (Lundbye-Jensen et al., 2011; Lauber et al., 2013b). We argue that the counterbalanced test order design is favorable for the present study, but test order effects may nevertheless indeed have contributed to the observed variability between participants.

In the present study, augmented feedback during motor practice was provided as a green cursor displaying the endpoint of the wrist flexion movement or force, respectively, in each trial. Feedback was provided at the end of each trial for reinforcement as opposed to other experimental models where continuous visual feedback is often provided. We chose to only provide visual endpoint feedback to promote the role other intrinsic feedback mechanisms including proprioception during the trial. Furthermore, Havas et al. (2021) have recently suggested that this can promote cognitive strategy use during motor learning compared to when feedback is presented continuously.

We tested how the augmented visual feedback influenced performance and found that when the augmented feedback was removed after the last practice block error increased in the post test for both training groups and tasks. This demonstrates that both the PC and FC group displayed positive effects of the augmented, visual feedback on endpoint performance during motor practice. Nevertheless, it is important to assess performance in the absence of augmented feedback following motor practice to allow assessment of flexibility, avoid feedback-dependent effects and to assess learning in agreement with the learning-performance distinction (Kantak and Winstein, 2012).

### Position control motor practice leads to larger increases in corticospinal excitability

The results from the supplementary experiment demonstrated that corticospinal excitability increased immediately following position control motor practice and 20 min later as evidenced by a left and upward shift of the TMS recruitments curve. While MEP amplitudes seemingly also increased following force control motor practice, this change was not significant, and the increase was significantly larger following position control practice as compared to force control motor practice. In other words, the results demonstrate that a single session of dynamic motor practice with an emphasis on position control leads to larger increases in CSE compared to isometric motor practice.

The facilitation of MEP amplitudes accompanying motor learning is supported by evidence from multiple previous studies (e.g., Lotze et al., 2003; Christiansen et al., 2017, 2018, 2020). It further supports previous findings of acutely increased CSE with the practice of both visuomotor (Perez et al., 2004; Cirillo et al., 2011), ballistic (Muellbacher et al., 2002; Carroll et al., 2008), and sequential (Pascual-Leone et al., 1994) motor tasks.

The increased response to magnetic stimulation likely reflects processes associated with motor learning when skill automaticity is low since the effects are marked during early motor practice (Cirillo et al., 2011; Christiansen et al., 2018) and motor practice on the fourth and fifth days of motor practice have been found not to be associated with acute increases in CSE (Rosenkranz et al., 2007; Christiansen et al., 2018). It is likely that within-session changes mark top-down processes such as attention and motivation leading to successful learning, and indeed Mawase et al. (2017) demonstrated that participants who successfully learned a skilled task demonstrated previous experiments have demonstrated greater use-dependent M1 or corticospinal plasticity compared to participants who performed the same amount of practice but did not accumulate learning.

In the present study isometric force control motor practice led to non-significant increases in CSE. Previous studies have, however, demonstrated increased CSE following both dynamic (Christiansen et al., 2020) and isometric (Christiansen et al., 2018) accuracy motor practice. Increased CSE has also been observed following practice of a discrete reinforcement accuracy motor task corresponding to the present study (Wiegel and Leukel, 2020). For MEPs obtained in different states Pearce and Kidgell (2010) observed increased amplitudes *during* performance of both position and force visuomotor accuracy tracking while Sugawara et al. (2013) found increased MEPs following force control practice but only during static contraction. This demonstrates, that changes in CSE can depend both on state and task type. In the present study, CSE was however assessed at rest prior to and following motor practice.

It may be that the lack of significant increases in CSE following the isometric motor practice in part could relate to the sample size and interindividual variability. A similar sample size was, however, sufficient to observe significant increases in CSE following dynamic motor practice. It may also be that isometric motor practice could have led to fatigue since contraction levels for some targets were higher in the force task compared to the position task and this could have led to potential fatigue. Fatigue would be expected to lead to acute decreases in MEP amplitudes at rest (Brasil-Neto et al., 1994) potentially due to an increase in the excitability of inhibitory intracortical circuits in M1 controlling corticospinal outputs (Seifert and Petersen, 2010). Differential demands for the position control task and the force control task could thus have led to different after-effects on CSE and thus on evoked MEP response amplitudes (Bradley et al., 2022). We argue, that the participants engaged in the two tasks with comparable effort due to the design of the task with both lower and higher force demands. The task design does, however, have limitations and differences between the two tasks concerning required force levels could have been diminished if the force needed to displace the lever during the position control task had been accurately adjusted relative to the MVC of each participant and set to match target 3 in the force task (i.e., 15% MVC). Due to the time between trials and the breaks between motor training and electrophysiological test procedures we find it unlikely that fatigue could explain the lack of increase in CSE following isometric motor practice in comparison to the increase observed following position control practice.

An additional limitation of the study design, which should be mentioned is that the absence of augmented endpoint feedback during baseline and post tests compared to practice sessions could contribute to increased variability in the performance measures due to lack of guidance and potentially decreased attention. Nevertheless, the results demonstrate distinct behavioral effects. Furthermore, it should also be acknowledged that while the results were relatively clear for overall behavioral effects across all targets, the results for specific targets are less clear. This likely relates to the lower number of trials performed for each individual target which is likely to influence the sensitivity of this analysis.

Following dynamic motor practice with an emphasis on position control, CSE increased significantly and significantly more compared to force control motor practice. This finding is interesting in itself, and it is also interesting that the larger increase in CSE was paralleled by more widespread positive behavioral effects accompanying dynamic motor practice. Rosenkranz and Rothwell (2012) previously demonstrated that proprioceptive inputs influence intracortical inhibition in the primary motor cortex and that modulation of proprioceptive integration in the motor cortex shapes human motor learning. We speculate that the distinct effects of dynamic motor practice observed in the present study may at least in part relate to richer information processing relating to requirements for both position and force control during dynamic motor practice and the more complex integration of peripheral sensory feedback during dynamic compared to isometric motor practice leading to more pronounced increases in CSE.

## Conclusion

In conclusion, dynamic motor practice of a discrete accuracy task with an emphasis on position control leads to improved movement accuracy and improved force control. Isometric motor practice improves force control but not movement accuracy. The two types of motor practice thus led to distinct behavioral effects with more pronounced improvements and positive between-task transfer effects accompanying dynamic motor practice. Furthermore, the results demonstrated that position control motor practice leads to bigger increases in corticospinal excitability compared to isometric force control motor practice. To our knowledge, this is the first study demonstrating these distinct effects on changes in corticospinal excitability. Therefore, the results provide novel insights into the behavioral and neural effects of position vs. force control motor practice. The results highlight the importance of dynamic motor practice with an emphasis on position control and this can be relevant to consider in other contexts of motor practice including neurorehabilitation training to improve behavioral outcomes and promote the underlying neuroplasticity.

## Data availability statement

The raw data supporting the conclusions of this article will be made available by the authors, without undue reservation.

## Ethics statement

The studies involving human participants were reviewed and approved by the Regional Ethics Committee for the Greater Copenhagen Area, Denmark (H-17019671). The patients/participants provided their written informed consent to participate in this study.

## Author contributions

MN, PW, and JL-J planned the experiments. PW and JL-J established the computerized task setup. MN, JB, and AN performed the experiments and analyzed the data. MN wrote the initial draft of the manuscript. All authors contributed to writing the final version of the manuscript.
